# Comparison of modified total leaflet preservation, posterior leaflet preservation, and no leaflet preservation techniques in mitral valve replacement – a retrospective study

**DOI:** 10.1186/s13019-019-0918-7

**Published:** 2019-06-07

**Authors:** Yilong Guo, Shuwu He, Tianguang Wang, Zelun Chen, Yue Shu

**Affiliations:** Department of Cardiovascular Surgery, The Second Affiliated Hospital of Hainan Medical University, 48th of Bai Shui Tang Road, Haikou, Hainan 570311 People’s Republic of China

**Keywords:** Mitral valve, Operative procedures, Heart valves

## Abstract

**Background:**

Mitral valve replacement with the total leaflet preservation technique can yield good results; however, its development is limited by patient-valve mismatch. Therefore, we compared the efficacies of the modified total leaflet preservation technique, posterior leaflet preservation technique, and no leaflet preservation technique in mitral valve replacement.

**Methods:**

Clinical records and echocardiographic data of 180 patients who underwent mitral valve replacement for rheumatic mitral valve disease between 2009 and 2017 were analysed retrospectively to summarise the operative experience and short-term (six months) results. The patients were divided into three groups: group A (*n* = 62), treated with the modified total leaflet preservation technique; group B (*n* = 80), treated with the posterior leaflet preservation technique; and group C (*n* = 38), treated with the no leaflet preservation technique.

**Results:**

No significant difference in the preoperative clinical data was noted between the groups (*p* > 0.05). The clamp and recovery times of group A were longer (*p* < 0.05) and shorter (*p* < 0.05), respectively, than those of groups B and C. The postoperative left ventricular end-diastolic diameter, left ventricular end-systolic diameter, and left ventricular ejection fraction of group A were significantly better than those of groups B and C. The incidence of low cardiac output syndrome in group A was lower than that in group C (*p* < 0.05). There was no postoperative left ventricular posterior wall rupture or mechanical valve dysfunction in group A.

**Conclusions:**

The short-term results of the modified total leaflet preservation technique were better than those of the other techniques. This technique is also suitable for patients with rheumatic mitral valve stenosis.

## Background

Mitral valve replacement (MVR)—an important treatment for rheumatic mitral valve disease—has been widely promoted and rapidly developed worldwide. Various MVR techniques have emerged. In 1964, Lillehei et al. found that the mortality and complications were lower, and the cardiac function was better if some part of the mitral valve was preserved through MVR [1]. Therefore, studies on valve preservation techniques have increased since then. Although Alsaddique proved that MVR with total leaflet preservation technique can achieve better results [2], its development has been limited by patient-valve mismatch. Therefore, we developed a modified version of this technique and compared its efficacy with that of the posterior leaflet preservation and no preservation techniques.

## Methods

### Patients

A total of 380 patients underwent valve replacement for rheumatic valve disease in our hospital between June 2009 and June 2017. The inclusion criteria were: mitral valve disease as the main diagnosis and rheumatic disease confirmed by pathological evaluation. The exclusion criteria were: patients with other severe cardiac diseases, such as valve disease or coronary artery disease, requiring concurrent surgical treatment. Finally, 180 patients were included in this retrospective study. The patients were divided into three groups: group A included 62 patients treated with the modified total leaflet preservation technique; group B included 80 patients treated with the posterior leaflet preservation technique; and group C included 38 patients treated with the no leaflet preservation technique. TEE (transoesophageal echocardiography) was performed at the end of the procedure in all patients to check for prosthesis dysfunction or left ventricular outflow tract stenosis (LVOTS).

### Operative methods

All the surgeries were performed by a single surgeon. The surgeries were performed via median sternotomy under cardiopulmonary bypass (CPB), mild hypothermia, and cardioplegic arrest. Ice water was placed in the pericardial cavity after cardiac arrest. The left atrium and mitral valve were exposed through the right atrial and atrial septal incisions, respectively. The leaflets and subvalvular structures were explored carefully, and valvuloplasty was performed as a priority, if suitable. If not, MVR was considered.

**Fig. 1:**
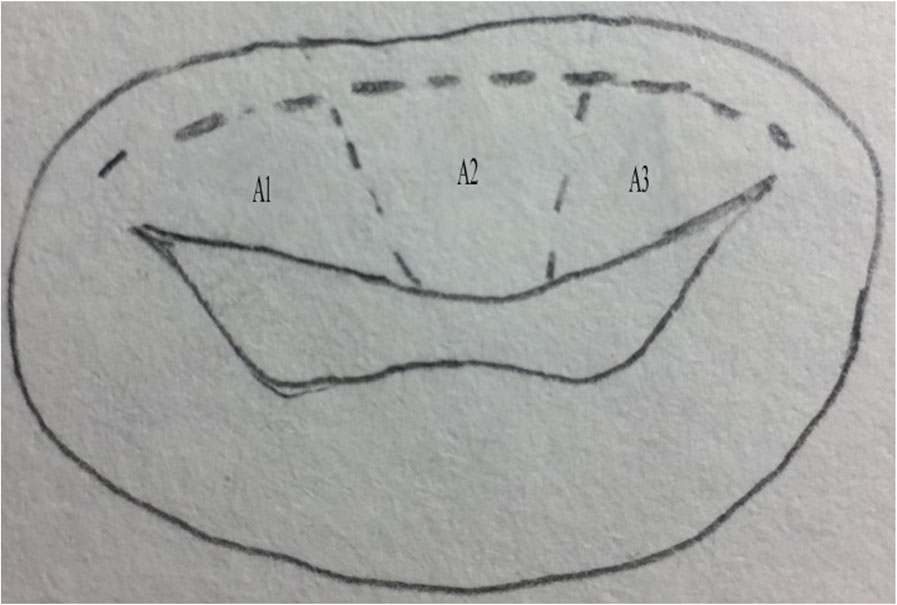
Anterior leaflet resection; A1, A2, and A3 are different parts of the anterior leaflet (Carpentier Type). A2 is removed while A1 and A3 are cut and trimmed. The surgical incision is shown by the dotted line

**Fig. 2:**
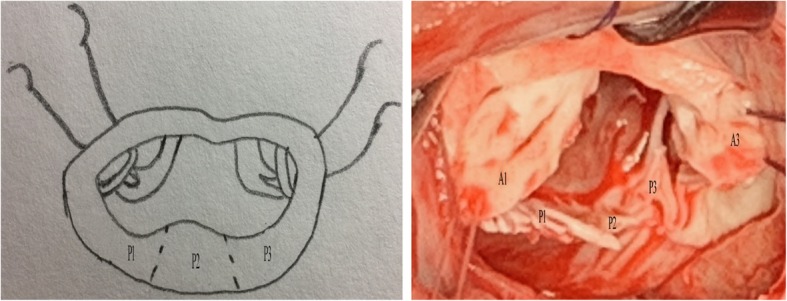
Anterior leaflet re-fixation and posterior part resection. P1, P2, and P3 are different parts of the posterior leaflet (Carpentier Type). The preserved anterior leaflet (A1 and A3) is fixed to the annulus junction separately. P2 is removed. The surgical incision is shown by the dotted line

**Fig. 3:**
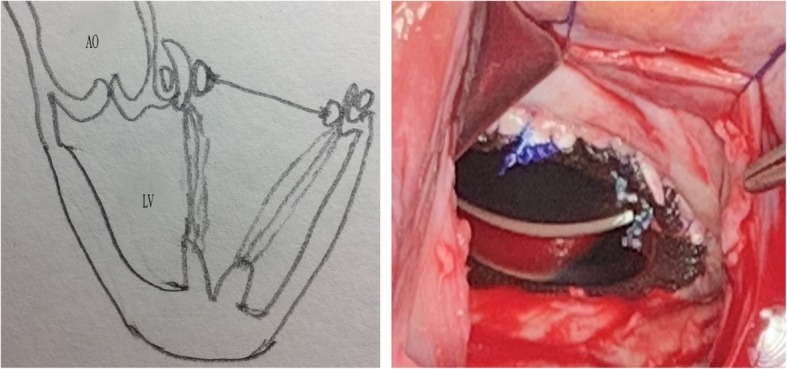
The preserved tissues are placed between the mechanical valve and the original annulus. Mechanical mitral valve implantation. AO, aorta; LV, left ventricle

Modified Total Leaflet Preservation Technique: The anterior leaflet was cut 2–3 mm away from the annulus. The leaflet and subvalvular structures of A2 (Carpentier Type) were resected (Fig. 1). The leaflet and subvalvular structures of A1 and A3 were trimmed (mainly, the thickened and calcified parts were resected), and only the tissue connected to the main chordae tendineae was preserved. Finally, the preserved leaflets of A1 and A3 looked like two buttons. They were re-attached to the original MV annulus, near the anterolateral and posteromedial commissures, respectively, with 2–0 pledget-supported Ticron sutures that were taken from the atrial to the ventricular side of the leaflet and slightly away from the free margin of the annulus. The most important point was to maintain appropriate tension in the leaflet and subvalvular tissue (Fig. 2); the leaflet and subvalvular structures of P2 (Carpentier Type) were resected. The leaflet and subvalvular structures of P1 and P3 were trimmed as described for A1 and A3 (Fig. 2). The mitral annulus was measured and a suitable mechanical valve was chosen; A SORIN medical prosthesis (SORIN GROUP, Mirandola, Italy) was used in all patients. When suturing near the commissure of the annulus, the preserved parts of A1 and A3 were fixed to the prosthesis valve ring, and the preserved tissues were clamped between the mechanical valve and the original annulus (Fig. 3). The thickened and calcified parts of the leaflet were removed as much as possible, and the thickened chordae tendineae were thinned. If the chordae tendineae were thickened, fused, calcified, and shortened in a relatively severe manner, tenolysis was performed first. Subsequently, some part of the leaflet was pruned and retained so that the chordae tendineae could be replaced and its length could be extended, thereby reducing the tension of the chordae tendineae and papillary muscle. However, if the lesion of the chordae tendineae was too severe to be preserved, the chordae tendineae were replaced with an artificial substitute.

Posterior Leaflet Preservation Technique: The anterior leaflet and subvalvular tissues were removed. The posterior leaflet and subvalvular structures were preserved (calcified tissue was removed). The margin of the posterior leaflet was folded if it was too long [3].

No Leaflet Preservation Technique: Both the leaflets and subvalvular tissues were removed before MVR.

### Data collection and processing

Preoperative data collected included the diagnosis, cardiac function (New York Heart Association, NYHA, grade), left ventricular end-diastolic dimension (LVEDD), left ventricular end-systolic dimension (LVESD), and left ventricular ejection fraction (LVEF). Peri-operative data on the clamp time, CPB time, recovery time, and early postoperative complications were also collected. LVEDD, LVESD, and LVEF were evaluated in every patient using Doppler echocardiography at the 6-month follow-up.

### Statistical analysis

Continuous data were expressed as mean ± standard deviation (SD) and compared using one-way analysis of variance. The least significant difference test was used for parametric variables, and the Welch and Dunnett’s T3 tests were used for non-parametric variables. The chi-square test was used to compare categorical variables. All statistical data were analysed using SPSS 19.0 (IBM Inc., Armonk, NY), and *p* < 0.05 was considered statistically significant.

## Results

### Preoperative information

There were no significant differences in the sex ratio, age, body surface area, major diagnosis, preoperative cardiac function (NYHA), LVEDD, LVESD, or LVEF between the three groups. The clinical profile of each group is shown in Table 1.

**Table 1:** Clinical profiles of the three groups of patients

	Group A (*n* = 62)	Group B (*n* = 80)	Group C (*n* = 38)	*p* value
Female sex	39 (63%)	44 (55%)	22 (58%)	0.637
Mean age ± SD y	54.61 ± 8.871	52.64 ± 8.570	51.53 ± 7.062	0.168
Body surface area (m^2^)	1.60 ± 0.11	1.59 ± 0.12	1.56 ± 0.12	0.228
Diagnoses				0.637
MS	28	32	12	–
MI	20	30	18	–
MS + MI	14	18	8	–
Heart function (NYHA)				0.779
II	10	18	6	–
III	40	46	24	–
IV	12	16	8	–
LVEDD (mm)	54.68 ± 6.83	55.58 ± 8.57	56.42 ± 5.88	0.404
LVESD (mm)	45.39 ± 3.91	45.79 ± 7.30	47.21 ± 5.53	0.214
LVEF (%)	56.66 ± 5.05	56.93 ± 4.27	57.45 ± 4.09	0.700

*LVEDD* left ventricular end-diastolic dimension, *LVEF* left ventricular ejection fraction, *LVESD* left ventricular end-systolic dimension, *NYHA* New York Heart Association, *MI* mitral valve insufficiency, *MS* mitral valve stenosis, *MS+ MI* mitral valve stenosis and mitral valve insufficiency

### Peri-operative data

Intra-operative Data: The clamp time of the three groups was significantly different, in the following order: group A > group B > group C (*p* < 0.05). The recovery time of the three groups was also significantly different, in the following order: group A < group B < group C (*p* < 0.05). The CPB time of group A was longer than that of group B (*p* = 0.009), and the CPB time of group B was shorter than that of group C (*p* = 0.001); however, there was no significant difference between the CPB times of groups A and C (*p* = 0.365). No significant difference in the prosthesis size was noted between the groups (*p* = 0.224). The pressure gradient through the prosthesis of group C was lower than that of groups A and B (*p* < 0.01), but there was no significant difference in the pressure gradient through the prosthesis between group A and group B (*p* = 0.656). The pressure gradient through the prosthesis indicated that no severe prosthesis dysfunction occurred in any of the groups (severe prosthesis dysfunction was defined as a pressure gradient of higher than 10 mmHg through the prosthesis). The operative data are shown in Table 2.

**Table 2:** Intra-operative data of the three groups of patients

	Group A	Group B	Group C	*p* value
Clamp time (min)	56.81 ± 4.31^a^	49.19 ± 3.33^a^	39.47 ± 3.70^a^	< 0.001
Recovery time (min)	22.06 ± 3.93^a^	27.59 ± 3.58^a^	39.47 ± 2.18^a^	< 0.001
CPB time (min)	79.80 ± 4.94^a^	76.90 ± 3.95^a.b^	79.74 ± 4.62^b^	0.002
Prosthesis size (mm)	26.60 ± 0.88	26.33 ± 1.10	26.58 ± 1.06	0.224
Pressure gradient (mmHg)	4.71 ± 0.88^a^	4.64 ± 1.05^b^	3.66 ± 0.88^a.b^	< 0.001

*CPB time* cardiopulmonary bypass time

Statistical comparisons: ^a^ *p* < 0.05; ^b^ *p* < 0.01

Short-term Complications: After six months of follow-up, the operative mortality was zero, and there was no incidence of infective endocarditis or prothesis dysfunction in all the three groups. The incidence of low cardiac output syndrome (cardiac index < 2 L•m^− 1^•m^− 1^) in group A was lower than that in group C (*p* = 0.011). There was no left ventricular posterior wall rupture or mechanical valve dysfunction in group A. The short-term complications are shown in Table 3.

**Table 3:** Short-term complications in patients of the three groups

	Group A (*n* = 62)	Group B (*n* = 80)	Group C (*n* = 38)	*p* value
Bleeding	1	2	1	0.921
Left ventricular rupture	0	1	2	0.127
Low cardiac output syndrome	1^a^	3	6^a^	0.007
Mechanical valve dysfunction	0	1	0	0.533
Pneumonia	5	10	8	0.167
Renal failure	1	1	3	0.096
Death	0	0	0	–
Infective endocarditis	0	0	0	–

Low cardiac output syndrome was defines as cardiac index less than 2 L•min^−1^•m^−1^

Statistical comparison: ^a^ *p* < 0.05

### Follow-up data

In groups A and B, LVEDD and LVESD had improved 6 months after the surgeries (*p* < 0.05). In group C, LVESD had improved (*p* = 0.007) 6 months after the surgeries, while LVEDD had not (*p* = 0.176). The LVEDD, LVESD, and LVEF of group A were significantly improved at 6 months after the surgery when compared with those of the other two groups (*p* < 0.05). The echocardiography characteristics are shown in Table 4.

**Table 4:** Echocardiography characteristics of the patients of the three groups

	LVEDD (mm)		LVESD (mm)		LVEF (%)	
	Preoperative	Postoperative	Preoperative	Postoperative	Preoperative	Postoperative
Group A	54.68 ± 6.83	50.10 ± 2.86^a^	45.39 ± 3.91	38.74 ± 2.50^a^	56.66 ± 5.05	57.92 ± 1.99^a.b^
Group B	55.58 ± 8.57	51.66 ± 2.84^a^	45.79 ± 7.30	41.05 ± 4.90^a^	56.93 ± 4.27	56.76 ± 2.24^b^
Group C	56.42 ± 5.88	55.03 ± 2.24^a^	47.21 ± 5.53	44.45 ± 2.63^a^	57.45 ± 4.09	55.55 ± 2.39^a^
*p* value	0.404	< 0.001	0.214	< 0.001	0.700	< 0.001

*LVEDD* left ventricular end-diastolic dimension, *LVEF* left ventricular ejection fraction, *LVESD* left ventricular end-systolic dimension

Statistic comparisons: comparison between groups, ^a^ *p* < 0.01; ^b^ *p* < 0.05

## Discussion

The main findings of this study were: 1) the short-term effects of the modified total leaflet preservation technique were superior to those of the other techniques; 2) the modified technique was suitable for different types of rheumatic mitral valve disease; and 3) since there is no definite indicator to determine the appropriate tension in a clinical setting and it mainly depends on the experience of the surgeon, only experienced doctors should perform this technique.

The mitral valve is a complex and well-coordinated anatomical structure whose integrity plays a key role in maintaining normal left ventricular function. The leaflets, annulus, chordae tendineae, papillary muscle, partial left atrial wall, partial left ventricular wall, and adjacent aortic annulus are the basic structures of the mitral valve. During systole, the mitral valve and subvalvular apparatus could lead to the movement of the annulus toward the apex and the concentric contraction of the left ventricle, thereby improving the ejection function of the left ventricle [4, 5]. Studies have demonstrated that the integrity of the mitral valve also plays a key role in maintaining right ventricular function [6]. After Lillehei et al. reported good results with the posterior leaflet preservation technique in 1964 [1], subsequent studies demonstrated that the anterior leaflet and subvalvular tissue are equally important in protecting the left ventricular function when compared to the posterior leaflet [7, 8]. Gomes conducted a study of both leaflet preservation techniques [9]. However, LVOTS is a serious complication that has restricted the development of the total leaflet preservation technique. A reduction in the left ventricular volume and excessive preservation of the anterior leaflet and subvalvular tissue are the main reasons for LVOTS [10, 11].

Alsaddique’s total leaflet preservation technique had two major drawbacks. One, the preserved tissue was extensive because it was restored to the annulus after the leaflet was cut from the centre and trimmed properly, and two, the technique only applied to patients with mitral regurgitation [2]. Compared to the traditional technique, the modified technique has two major improvements. One, the leaflet and subvalvular tissues of A2 and P2 were resected and the other part was trimmed appropriately, resulting in reduction of the preserved tissues. Only the tissues connected to the main chordae tendineae were preserved in a button-like manner and fixed at the junction of the annulus. Two, the modified technique is also considered suitable for rheumatic mitral stenosis.

Our modified technique resulted in the following major results: 1) the incidence of low cardiac output syndrome was reduced; 2) LVEDD, LVESD, and LVEF were significantly improved postoperatively; and 3) the modified technique was more complicated and the clamp time was longer; therefore, myocardial ischaemic time was increased. However, the recovery time was shorter. The recovery time is an important index for resuscitation effects; therefore, the resuscitation effects were better in group A.

In our experience, the following points should be focused on when applying this modified technique. One, the anterior leaflet and subvalvular tissue must be managed carefully. Excessively preserved tissues might protrude into the left ventricular outflow tract and lead to LVOTS [11]. Therefore, the leaflet of the A2 area was resected routinely and the remaining tissue connected to the main chordae tendineae was trimmed and preserved in a “button-like” manner. Subsequently, the preserved tissue of the anterior leaflet was transferred and fixed at the junction of the annulus. The chordae tendineae and papillary muscles were kept in a suitable tension. This was beneficial not only for preserving the integrity of the mitral valve but also for protecting left ventricular function [9, 12]. Two, the preserved tissue must be placed between the mechanical valve and the original annulus to reduce its effects on the mechanical valve [3]. Three, only the main chordae tendineae were preserved. If the lesion of the chordae tendineae was too severe to be preserved, the chordae tendineae could be replaced with an artificial substitute [13]. MVR with leaflet preservation technique may result in patient-valve mismatch, especially in female patients with severe mitral valve stenosis [14]. The preserved valve and subvalvular apparatus should be trimmed to the greatest extent. If a suitable valve still cannot be implanted, then the total leaflet preservation technique should be abandoned, and the posterior leaflet preservation technique or the no valve preservation technique should be applied.

This study has some limitations. This was a retrospective, non-randomised study with no multivariate analysis performed; therefore, a certain selection bias exists. Further prospective, randomised, large-scale, long-term studies with multivariate analysis are required to validate our findings. Moreover, there was a larger number of patients with mitral regurgitation in group C. This could justify the slight decrease of LVEF in this group, so more attention should be paid to this issue in future studies.

## Conclusions

In conclusion, the short-term results of the modified total leaflet preservation technique were better than those of the other techniques. This modified technique is also suitable for patients with rheumatic mitral valve stenosis.

## Abbreviations

CPB: Cardiopulmonary bypass
LVEDD: Left ventricular end-diastolic dimension
LVEF: Left ventricular ejection fraction
LVESD: Left ventricular end-systolic dimension
LVOTS: Left ventricular outflow tract stenosis
MI: Mitral valve insufficiency
MS: Mitral valve stenosis
MVR: Mitral valve replacement
NYHA: New York Heart Association
SD: Standard deviation
TEE: Transoesophageal echocardiography
